# Comparative Evaluation of Drying Methods for Vegetable Waste Aimed at Producing Natural Functional Food Ingredients

**DOI:** 10.3390/molecules31071190

**Published:** 2026-04-03

**Authors:** Tamara Tultabayeva, Umyt Zhumanova, Kadyrzhan Makangali, Assem Sagandyk, Aknur Muldasheva, Aruzhan Shoman, Mukhtar Tultabayev

**Affiliations:** 1 International School, University of California, Davis, 1 Shields Ave, Davis, CA 95616, USA; 2 Department of Food Technology and Processing Product, S.Seifullin Kazakh Agrotechnical Research University, Astana 010000, Kazakhstan; 3 Scientific and Innovation Center Agro Tech, Astana IT University, Astana 010000, Kazakhstan; 4 Technology and Standardization Department, Kazakh University of Technology and Business of K.Kulazhanov, Astana 010000, Kazakhstan

**Keywords:** vegetable by-products, drying methods, bioactive compounds, functional food ingredients, waste valorization, vacuum-microwave drying

## Abstract

This study presents a comparative evaluation of four drying methods for carrot, red beet, and pumpkin pomace to produce natural functional food ingredients. The work addresses the valorization of 35–45% vegetable processing waste—a rich source of bioactive compounds—aligning with circular bioeconomy principles and Kazakhstan’s goals for deep processing of agricultural raw materials. The compared methods were convective drying (CD), ultrasound pretreatment + convective drying (US + CD), vacuum-microwave drying (VMD), and ultrasound pretreatment + vacuum-microwave drying (US + VMD). Drying kinetics, water activity, physicochemical and functional properties of powders, retention of bioactive compounds, color characteristics, thermal stability, and sensory attributes were assessed. Kinetics were fitted using Midilli et al., Page, and Weibull models. US + VMD provided the highest drying acceleration (6–11 times faster than CD), reaching final moisture of 5.1–5.9%, water activity a_w_ 0.27–0.31 in 80–170 min, and bioactive compound retention of 90–95% (carotenoids 92–95%, betalains 90–94%). It also delivered superior flowability (Carr’s index 22.5–30.4%), dispersibility (80–88% in 30 s), and thermal stability (75–85% at 200 °C). Acceleration varied by raw material: maximum for beet (up to 11×) due to soluble sugars and nitrates, minimum for pumpkin (5.5–8×) due to dietary fibers and pectins, and intermediate for carrot (6–9×) influenced by carotenoids’ dielectric properties. The results highlight US + VMD’s strong potential for producing functional powders to replace synthetic additives in food systems. Effective method selection and parameter optimization require consideration of raw material type and rheological characteristics.

## 1. Introduction

Vegetable processing waste (pomace from carrot, red beet, and pumpkin) accounts for 35–45% of the initial raw material mass and represents a significant source of bioactive compounds: β-carotene (65–210 μg/g fresh weight in carrots), betalains (up to 241 mg/100 g dry matter in beets), carotenoids, polyphenols, and dietary fibers (up to 55–60% in carrots and pumpkins) [1,2,3,4]. These compounds exhibit proven antioxidant, anticancer, cardioprotective, and anti-inflammatory activities; however, pomace is predominantly used as animal feed or disposed of in landfills, resulting in resource losses and environmental damage [5,6].

Convective hot-air drying (CD) remains the dominant method due to its low cost, but it causes significant losses of thermolabile compounds: 30–50% of carotenoids in carrots, 40–60% of betalains in beets, and a reduction in the antioxidant capacity of pumpkins due to oxidation and thermal degradation [7,8,9,10]. This is accompanied by color changes (ΔE > 15–20), high water activity (a_w_ 0.39–0.46), poor flowability (Carr’s index > 32), and low dispersibility. Freeze-drying preserves bioactive compounds at 96–98%, but energy costs are 3–5 times higher and the process lasts 24–72 h, limiting scalability [11,12].

Vacuum-microwave drying (VMD) combines volumetric heating with reduced pressure (5–15 kPa), lowering the product temperature to 30–60 °C and reducing drying time by 5–10 times compared to CD [13,14,15]. This ensures retention of carotenoids and betalains at 90–95%, final moisture content of 5–6%, a_w_ 0.28–0.33, and improved powder properties (Carr’s index 23–30, dispersibility 72–88% within 30 s, thermal stability up to 200 °C) [16,17]. Despite the high efficiency of microwave heating, the method is not without drawbacks. The main limitations include non-uniform volumetric heating [18], intense vapor evolution that exceeds the capabilities of the vacuum system [19], and high capital and energy costs [20]. These limitations are particularly evident when drying vegetable crops with high contents of thermolabile pigments, such as pumpkin, carrot, and beet. In this regard, the present study proposes the application of preliminary ultrasound pretreatment (US), aimed at eliminating the aforementioned drawbacks by improving mass and heat transfer, enhancing drying uniformity, and providing additional protection for bioactive compounds. One of the methods for overcoming the aforementioned drawbacks is ultrasonic pretreatment, which enhances the effect through cavitation: it creates microchannels, reduces particle size, accelerates drying by 30–70%, and increases the retention/preservation of bioactive compounds by 10–15% [21,22,23].

Despite individual studies on VMD and US for carrots [24], beets [25], or pumpkins [26], no systematic comparisons of the four schemes (CD, US + CD, VMD, US + VMD) across all three types of pomace have been conducted simultaneously. Most works are limited to drying kinetics or individual bioactive compounds, without a comprehensive evaluation of functional-technological properties (flowability, hydration, creaminess, thermal stability) and applied potential in real food matrices (bread, sausages, ice cream, yogurts) [27,28,29]. There is also insufficient data on optimizing VMD parameters for each raw material type and the impact of the US + VMD combination on the final consumer characteristics of the powders.

This study aims to provide a comparative evaluation of drying methods for carrot, red beet, and pumpkin pomace to produce high-quality natural functional ingredients. The novelty lies in: (i) simultaneous comparison of four technological schemes across three raw material types; (ii) optimization of parameters (power, pressure, temperature) for maximum retention of bioactive compounds and functional properties; and (iii) integrated quality assessment (kinetic, physicochemical, bioactive, color, sensory, and thermal characteristics). Limitations of the work include laboratory scale, absence of long-term storage tests (>12 months), and pilot trials.

The obtained results justify the methods for sustainable valorization of vegetable waste into functional ingredients.

## 2. Results

This section presents the experimental results comparing four drying methods for carrot, red beet, and pumpkin pomace: convective drying (CD), ultrasound pretreatment followed by convective drying (US + CD), vacuum-microwave drying (VMD), and the combination of ultrasound pretreatment with vacuum-microwave drying (US + VMD). All data were obtained in triplicate and subjected to statistical processing (*p* < 0.05, Duncan’s test).

### 2.1. Drying Kinetics and Processing Time

Drying kinetics is a determining factor for process efficiency, energy consumption, and the quality of the final product (retention of carotenoids, betalains, powder texture). In this study, the dynamics of moisture removal from carrot, beet, and pumpkin pomace were investigated using four methods: convective drying (CD), ultrasound-assisted convective drying (US + CD), vacuum-microwave drying (VMD), and combined ultrasound pretreatment with vacuum-microwave drying (US + VMD). Key parameters for evaluating kinetics included: time to reach final moisture content < 6% (target for stable storage and low a_w_ ≤ 0.35), relative acceleration compared to CD, effective moisture diffusivity coefficient (D_eff_), and process phases (accelerated—free moisture; decelerated—bound moisture). Table 1 presents comparative data on drying time to <6% moisture and relative acceleration for all three pomace types.

**Figure 1:**
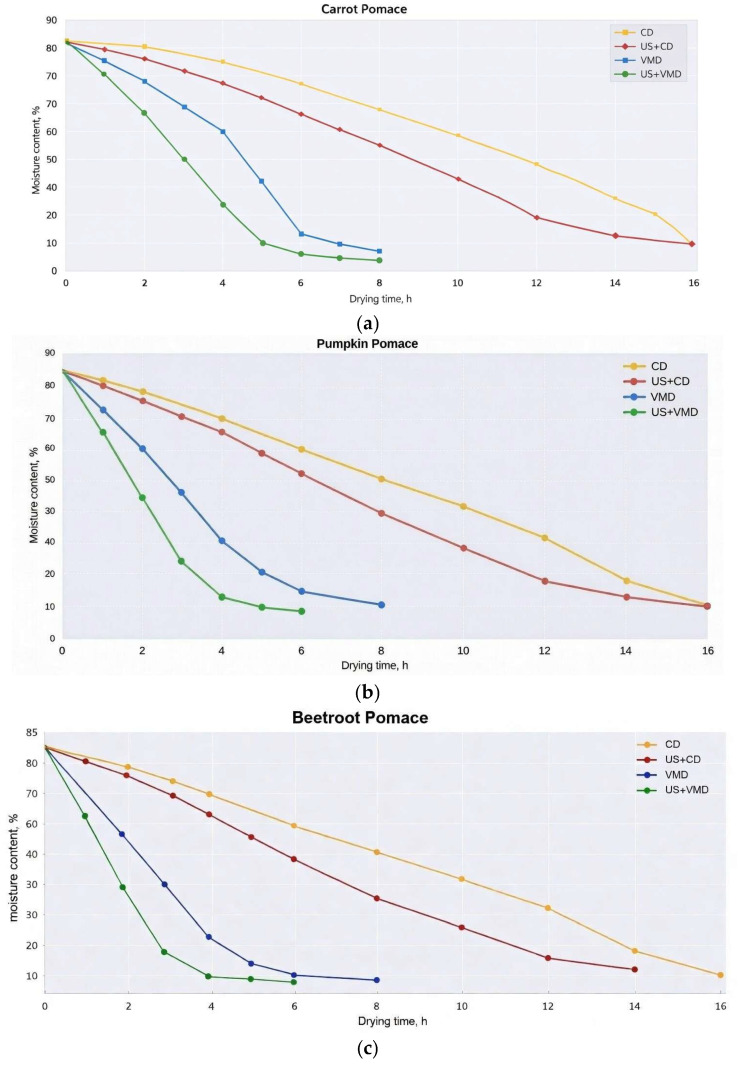
Changes in moisture content during drying for three types of cake using four drying methods. (**a**)—carrots; (**b**)—pumpkins; (**c**)—beets.

Analysis of the drying curves (Figure 1) shows that the combined methods (VMD and especially US + VMD) reduced drying time by 5–11 times compared to CD. For carrot pomace, the time to reach <6% moisture was 120–180 min with VMD and 90–140 min with US + VMD (versus 10–14 h with CD). A similar trend was observed for beet (VMD 100–160 min, US + VMD 80–130 min vs. CD 12–16 h) and pumpkin (VMD 140–200 min, US + VMD 110–170 min vs. CD 11–15 h). Ultrasound pretreatment reduced subsequent drying time by 30–50% due to cavitation, microchannel formation, and cell wall disruption, which enhanced mass transfer. It was found that the effective moisture diffusivity coefficient (D_eff_) under US + VMD was 3–5 times higher than under CD (data confirmed by Weibull and Midilli et al. models, R^2^ > 0.98).

The curves exhibit a typical decreasing exponential dependence with two distinct phases: an accelerated phase (up to 4 h—removal of free moisture) and a decelerated phase (beyond 4 h—removal of bound moisture). The steepest decline is observed for US + VMD (green curves): residual moisture < 10% is achieved within 4–8 h (depending on the vegetable type). VMD without pretreatment (blue curves) occupies an intermediate position, while CD (orange curves) is the most prolonged (up to 14+ h). Ultrasound pretreatment substantially accelerates both types of drying, but the synergistic effect is maximal specifically with VMD (volumetric heating + vacuum + microporosity induced by ultrasound).

#### Mathematical Modeling of Drying Kinetics

To describe the kinetics of dimensionless moisture content MR = M(t)/M_0_, five thin-layer models were tested: Page (MR = exp(−k·t^n^)), Midilli et al. (MR = a·exp(−k·t^n^) + b·t), Logarithmic (MR = a·exp(−k·t) + c), Henderson and Pabis (MR = a·exp(−k·t)), and Weibull. Parameters were determined using nonlinear regression (Origin/Pro, Levenberg–Marquardt algorithm).

The Midilli et al. model provided the best fit to the experimental curves across all methods (R^2^ = 0.998–0.9999; RMSE = 0.005–0.015; χ^2^ < 10^−4^), particularly for US + VMD (showing the highest nonlinearity). The Page model (R^2^ > 0.997) also effectively captured the acceleration in the initial phase under the influence of ultrasound pretreatment and microwave heating. For CD and US + CD, the Logarithmic and Henderson and Pabis models were adequate but inferior in fitting criteria.

It was established that model parameters depend on the drying method. For example, in US + VMD, the exponent “n” in the Page/Midilli models is higher (1.2–1.6), indicating acceleration due to cavitation and microchannel formation, while the rate constant “k” reaches its maximum in US + VMD (3–8 times higher than in CD), confirming the synergistic effect.

The obtained drying kinetics dependencies and critical parameters (time to <6% moisture, acceleration 5–11×, D_eff_ 3–5×) highlight the advantages of the combined US + VMD method for thermolabile vegetable waste. This necessitates further analysis of the relationship between moisture kinetics and water activity (a_w_) dynamics, as reducing moisture to <6% directly correlates with achieving a safe a_w_ level ≤0.35 and preserving bioactive compounds.

### 2.2. Changes in Water Activity During Drying of Beet, Carrot, and Pumpkin Pomace

**Figure 2:**
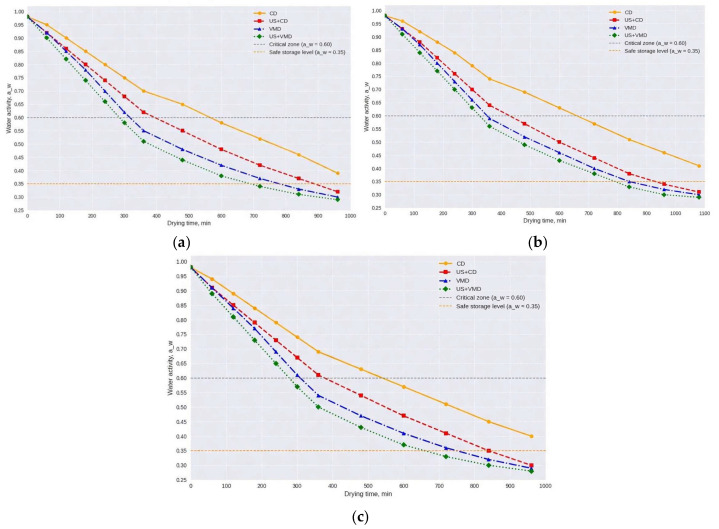
Changes in water activity (a_w_) during drying using different methods. (**a**)—carrots; (**b**)—pumpkins; (**c**)—beets.

Water activity (a_w_) is one of the key parameters determining microbiological stability, retention of bioactive compounds (particularly betalains), and the physicochemical properties of the final powder. In this study, the dynamic changes in a_w_ were investigated during drying of vegetable waste using different methods: convective drying (CD), ultrasound pretreatment followed by convective drying (US + CD), vacuum-microwave drying (VMD), and combined ultrasound pretreatment with vacuum-microwave drying (US +VMD). Figure 2 shows that the curves of a_w_ change from the initial value ≈ 0.98–1.00 (typical for fresh pomace with 80–90% moisture) down to final values of 0.25–0.35 within 900–1000 min.

All curves exhibit a nonlinear decreasing pattern with two distinct phases: rapid decline in the initial period (removal of free moisture) and slowing down at later stages (removal of bound moisture within cellular structures, capillaries, and complexes with sugars/pigments). Critical levels are noted: a_w_ = 0.60 (zone where growth of most microorganisms stops) and a_w_ = 0.35 (safe level for long-term storage, minimizing oxidation, enzymatic reactions, and powder caking). Analysis of the drying curves revealed significant differences between the methods. Convective drying (CD) is characterized by the gentlest (slowest) decline, whereas ultrasonic pretreatment substantially accelerates the process in US + CD (red curve). Vacuum-microwave drying (VMD) provided a significantly higher drying rate, promoting rapid boiling and removal of bound moisture while minimizing the time of exposure to oxygen. The combined method US + VMD demonstrates the highest drying kinetics. The synergistic effect of U and VMD ensured the most efficient process.

#### Mathematical Modeling of Water Activity Changes During Drying

To mathematically describe the kinetics of a_w_ reduction, adapted empirical models were tested: Page (a_w_ = exp(−k·t^n^) + c), Logarithmic (a_w_ = a·exp(−k·t) + c), Midilli et al. (a_w_ = a·exp(−k·t^n^) + b·t), and Weibull. Parameters were determined using nonlinear regression. The Midilli et al. model showed the best fit to experimental data across all methods (R^2^ = 0.998–0.9999, RMSE < 0.01), especially for US + VMD, where nonlinearity is most pronounced. The Page model (R^2^ > 0.997) effectively described acceleration in the initial phase under the influence of ultrasound pretreatment and microwave heating. For CD and US + CD, the Logarithmic and Weibull models were adequate. The rate constants “k” and nonlinearity exponent “n” were maximal in US + VMD (k 4–10 times higher than in CD), confirming the synergistic effect of the combination.

The dependencies indicate that water activity a_w_ (t) is a complex function of time, governed by the nonlinear relationship with moisture content through sorption/desorption isotherms, as well as water-binding processes (monomolecular layer, capillary water, interactions with sugars and pigments). Therefore, a detailed study of a_w_ reduction kinetics in combination with desorption isotherms and evaluation of their impact on betalain retention is necessary. This will enable optimization of parameters for combined drying methods and improvement of the quality and stability of the resulting powders.

### 2.3. Physicochemical Properties of Powders Obtained by Different Drying Methods of Vegetable Waste

It was established that a reduction in the average particle size (especially below 150 μm) positively correlated with improved functional properties: the Carr index decreased by 4–8%, dispersibility within 30 s increased by 8–15%, and wetting time was reduced by a factor of 1.4–1.9. Smaller particles (D50 < 100 μm) provided a larger specific surface area, accelerated rehydration, and enhanced creaminess in model systems. However, at particle sizes < 50 μm, increased dustiness and a greater tendency toward agglomeration were observed at relative humidity > 65%. The optimal range for functional properties was 70–140 μm (predominantly in powders produced by ultrasonic pretreatment + vacuum-microwave drying).

The results indicate that final moisture content and water activity (a_w_) were minimal for VMD and US + VMD (Table 2).

Moisture content ranged from 5.2–6.1% (VMD/US + VMD) compared to 7.4–8.2% (CD/US + CD). Water activity was in the range of 0.28–0.33 for VMD versus 0.39–0.46 for CD, significantly reducing microbiological risk and extending shelf life. Carr’s index (flowability indicator) was best for US + VMD (23.7–30.4%), indicating good flow properties, whereas for CD it exceeded 32–37%, indicating poor flowability and tendency to agglomerate. Dispersibility within 30 s reached 72–88% for VMD/US + VMD versus 59–71% for CD, and complete dissolution time was reduced by 1.5–2 times (45–68 s vs. 78–94 s). Values for US + VMD were statistically superior to all other methods (*p* < 0.05).

### 2.4. Retention of Bioactive Compounds in Powders Obtained by Different Drying Methods of Vegetable Waste

The results indicate that retention of thermolabile compounds was maximal for US + VMD (Table 3).

Carotenoid retention in carrot powder reached 92–95%, betalains in beet 90–94%, and total carotenoids in pumpkin 91–93%. With CD, losses were 23–27% (carotenoids) and up to 35% (betalains). US + CD improved retention by 10–15% compared to pure CD but was inferior to VMD by 15–20%. It was also found that antioxidant capacity (DPPH/FRAP) correlated with bioactive retention and was highest for US + VMD.

### 2.5. Color Characteristics and Thermal Stability of Powders Obtained by Different Drying Methods of Vegetable Waste

It was established that powder color was better preserved with VMD/US + VMD: ΔE ranged from 8–12 versus 18–25 for CD. «L» (lightness) was higher, «A» (redness for beet) and «B» (yellowness for carrot/pumpkin) were closer to fresh raw material. Thermal stability (residual bioactive activity after 30 min at 200 °C) reached 75–85% for US + VMD versus 45–60% for CD.

### 2.6. Sensory and Functional Properties of Powders Obtained by Different Drying Methods of Vegetable Waste

Studies showed that on a 9-point scale, US + VMD powders received the highest scores for creaminess (7.8–8.5), color (8.0–8.7), and overall acceptability. CD powders exhibited bitterness and reduced creaminess (3.9–5.7). VMD and especially US + VMD provided the best combination of process speed, bioactive retention, functional properties, and powder quality. CD was inferior in all key parameters despite its cost-effectiveness.

### 2.7. Data Processing

Two-way ANOVA confirmed a significant interaction between the type of raw material and the drying method for most parameters (F{6,24} = …, *p* < 0.001), which justifies separate discussion of the effects by vegetable type.

## 3. Discussion

The results confirm the superiority of the combined ultrasound pretreatment with vacuum-microwave drying technology (US + VMD) over traditional and intermediate methods in processing vegetable waste from carrot, beet, and pumpkin. The main advantages of US + VMD are manifested in three key aspects: process speed, retention of thermolabile bioactive compounds, and functional-technological properties of the final powders.

### 3.1. Drying Kinetics and Mass Transfer Efficiency of Water Activity

Vacuum-microwave drying (VMD) reduced process time five to nine-fold compared to convective drying (CD), while the combination of ultrasound pretreatment with VMD (US + VMD) provided an additional acceleration of 20–35%, resulting in an overall range of 6–11× relative to CD. These findings are consistent with literature data on vacuum-microwave drying of vegetable waste and pomace [13,21,27], where acceleration due to volumetric heating under reduced pressure (5–15 kPa) and low product temperature (30–60 °C) reaches 4–10×, and ultrasound pretreatment adds 20–50% through cavitation and microchannel formation. Ultrasound pretreatment increased the effective moisture diffusivity coefficient (D_eff_ 3–5 times higher than in CD), as confirmed by Weibull and Midilli et al.’s models (R^2^ > 0.98). At the same time, it was found that the drying acceleration effect is uneven across different raw materials. The maximum relative acceleration was observed for beetroot (up to 11×), which is likely due to its high content of soluble sugars and inorganic nitrates. These compounds reduce system viscosity, promote rapid evaporation of free moisture, and improve the dielectric properties of the material under vacuum-microwave heating conditions [16,25]. In pumpkin pomace, the relative acceleration was the lowest (5.5–8×), which can be explained by the high proportion of insoluble dietary fiber and pectic substances. These components form additional diffusion barriers and slow down the removal of bound moisture even when using combined intensive drying methods [26,30,31,32]. For carrot pomace, the effect was intermediate (6–9×). It is suggested that carotenoids (in particular β-carotene) exert a noticeable influence on local changes in the dielectric properties of the material during vacuum-microwave heating, although direct measurements of dielectric permittivity and loss factor were not performed in the present study [24,33].

These interpretations are consistent with literature data on the influence of compositional features of vegetable raw materials on drying kinetics in combined processes [13,21,27,34,35,36]. However, their final confirmation would require additional direct measurements of soluble sugar/nitrate content, fractional composition of dietary fiber, as well as dielectric characteristics (dielectric constant ε′ and loss factor ε″) of the pomace before and after pretreatment. Within the framework of the present study, these aspects were not analyzed. Therefore, the explanations should be regarded as working hypotheses based on the known physicochemical properties of the raw materials and the observed differences in drying kinetics.

US + CD improved kinetics by 30–50% compared to pure CD but significantly lagged behind VMD-based methods. This highlights that the main contribution to acceleration comes not only from pretreatment (microchannels and cavitation) but also from the synergy of the vacuum environment with volumetric microwave heating: reduced pressure lowered the boiling point, minimized temperature gradients and oxidation, while rapid vapor removal prevented case-hardening [22,28]. The critical limitation of CD remains diffusion restrictions during the falling rate period and surface heating, leading to prolonged exposure in the high-moisture zone (a_w_ > 0.60) and increased risk of enzymatic/oxidative processes.

The linkage between moisture kinetics and water activity (a_w_) dynamics further strengthened the advantages of combined methods: in US + VMD, a_w_ decreased most sharply, reaching the safe level ≤ 0.35 in the shortest time (80–170 min depending on the vegetable), thereby minimizing degradation of thermolabile compounds (betalains in beet, carotenoids in carrot/pumpkin). In CD, prolonged exposure in the critical a_w_ zone of 0.60–0.90 increased the risk of microbiological spoilage and pigment loss. However, VMD and US + VMD are not without drawbacks: intense vapor release in the initial phase may exceed vacuum system capacity (risk of pump overload), while heating nonuniformity at high power or thick layers may cause local overheating and quality heterogeneity (especially in pumpkin with high fiber content). Ultrasound pretreatment adds energy costs for pretreatment, although the overall gains in time and quality generally outweigh them.

Thus, analysis of drying kinetic curves and a_w_ changes for carrot, beet, and pumpkin pomace demonstrated that the combined US + VMD method provides the highest mass transfer rate, minimal process time, and most efficient reduction of water activity compared to CD, US + CD, and pure VMD. The synergistic effect (ultrasound disrupts cellular barriers and increases porosity, while VMD ensures volumetric low-temperature heating and rapid vapor removal) makes this approach the most promising for thermolabile pomace rich in bioactive compounds. At the same time, pure convective drying is characterized by the lowest kinetic efficiency due to pronounced diffusion limitations, surface heating, and prolonged exposure in the high a_w_ zone, making it the least preferable for industrial production of natural powders with maximum pigment retention (90–95%). The data emphasize the need for further optimization of parameters (microwave power, vacuum level, ultrasound time/intensity) considering raw material specifics to minimize nonuniformity and energy costs while preserving high product quality.

### 3.2. Retention of Bioactive Compounds and Thermal Stability

Bioactive compounds and antioxidant properties of the dried pomace, as presented in Table 3, show that vacuum-microwave drying (VMD and US + VMD) provided significantly higher retention of β-carotene (up to 90–95%) and antioxidant capacity compared to convective drying (CD), with ultrasound pretreatment further improving these parameters in the combined methods. These values significantly outperformed convective drying (CD), where losses reached 23–35%, consistent with known data on degradation of thermolabile pigments under prolonged hot air exposure and oxidation [8,9,10,23]. The advantage of US + VMD is due to the combination of low product temperature (30–60 °C), shortened exposure time, and minimization of local overheating thanks to uniform mass transfer through ultrasound-induced microchannels. Compared to pure VMD, the additional gain in retention was 10–15%, confirming the synergistic effect of pretreatment [24,36].

Thermal stability of the powders (residual bioactive activity after 30 min at 200 °C) reached 75–85% with US + VMD, 1.3–1.8 times higher than with CD (45–60%). This makes the resulting powders promising for incorporation into high-temperature technological processes (baking, extrusion, sterilization), where traditional methods typically lead to substantial loss of functionality [26,37]. However, even with US + VMD, pumpkin (high fiber content) showed slightly lower thermal stability compared to carrot and beet, related to structural heterogeneity and local overheating zones. Additionally, the data were obtained under laboratory conditions; long-term pigment stability during storage under real conditions (light, oxygen, humidity) requires further investigation.

### 3.3. Physicochemical and Functional Properties of Powders

Low final moisture content (5.1–6.1%) and water activity (a_w_ 0.27–0.33) with US + VMD ensured high microbiological stability and a predicted shelf life of 2–3 years without risk of mold growth, caking, or oxidation.

According to the literature on comparable dried vegetable powders (carrot, beetroot, pumpkin) with water activity (a_w_) between 0.28 and 0.35 and packaged in barrier materials, the expected shelf life under controlled storage conditions (20–25 °C, relative humidity < 60%, protection from light and oxygen) is 12 to 36 months [38,39]. Nevertheless, as no direct stability testing (real-time or accelerated) was carried out in the present study, this range should be regarded as approximate and requires validation through future long-term storage trials with regular monitoring of key quality attributes, including bioactive compound preservation, color, water activity (a_w_), and microbiological contamination, over at least 12–24 months.

In contrast, CD resulted in final a_w_ of 0.39–0.46, substantially increasing the probability of spoilage and bioactive degradation even under controlled storage conditions [9,40]. Carr’s index of 22.5–30.4% and dispersibility of 72–88% within 30 s indicate excellent flowability and rapid rehydration, which is critical for applications in dry mixes, yogurts, ice cream, sausage formulations, and other systems [35,41,42,43].

The highly porous structure formed by ultrasound cavitation and intense vacuum vaporization significantly improved water-holding capacity and sensory characteristics (mouthfeel creaminess 7.8–8.5 points on a 9-point scale). At the same time, in some cases with US + VMD (especially pumpkin), increased powder dustiness and tendency to agglomerate under high ambient humidity were noted, requiring optimization of grinding and packaging parameters. Furthermore, energy costs for ultrasound pretreatment and the vacuum system may limit the economic attractiveness of the method at small production scales.

### 3.4. Applied Advantages and Limitations

The US + VMD powders demonstrate clear applied advantages: effective replacement of synthetic colorants, stabilizers, and antioxidants in bakery products (delaying staling by 20–40% due to dietary fibers), meat products (emulsion stabilization, extended shelf life), dairy desserts (improved texture and stability of ice cream), and confectionery [10,42,44]. This fully aligns with the global trend toward “clean label,” natural ingredients, and circular bioeconomy principles (valorization of vegetable processing waste).

At the same time, the study has several limitations: laboratory scale, absence of long-term storage tests (>12 months), dependence of results on specific raw material varieties, maturity stages, growing conditions, and equipment characteristics. Heating nonuniformity and local overheating in VMD (especially at large loads) may lead to quality variability under industrial conditions. Economic assessment (capital investments, energy costs, ultrasound equipment cost) and pilot trials on real production remain necessary to confirm the scalability and competitiveness of the technology.

## 4. Materials and Methods

### 4.1. Raw Materials and Sample Preparation

Pomace from carrot (*Daucus carota* L., variety “Nantskaya”), red beet (*Beta vulgaris* L., variety “Bordo”), and pumpkin (*Cucurbita maxima* L., variety “Vitaminnaya”) was obtained after industrial juice pressing at a facility in the Almaty region (Kazakhstan). Fresh pomace was immediately transported to the laboratory at 4 ± 1 °C and stored for no longer than 12 h before processing. The initial moisture content of the pomace was 78–85% (carrot 82.5 ± 1.2%, beet 84.1 ± 1.0%, pumpkin 80.3 ± 1.5% according to AOAC method 934.01 [45]). The pomace was cleaned of large inclusions and uniformly spread in a layer 10–15 mm thick for all drying methods.

### 4.2. Ultrasound Pretreatment (US)

Ultrasound pretreatment was performed using an Hielscher UP400St ultrasonic homogenizer (Hielscher, Teltow, Germany) operating at 20–35 kHz and power of 200–600 W. Pomace (200 g) was placed in distilled water at a solid-to-liquid ratio of 1:3, and treated at an amplitude of 50–80% (power 300–500 W) for 10–20 min at 25 ± 2 °C, with 5-min intervals to prevent overheating. After treatment, the pomace was pressed and used for subsequent drying.

Ultrasonic pretreatment was performed in pulsed mode to prevent excessive heating and degradation of bioactive compounds. The total processing time was 15 min (three cycles of 5 min active ultrasonic treatment + 2-min pauses between cycles to cool the suspension down to 28–30 °C). Parameters of each cycle: frequency 24–28 kHz, amplitude 70–80% (corresponding to power 380–480 W), suspension volume 600 mL (200 g of press cake + 600 mL distilled water), initial temperature of each cycle 25 ± 2 °C. After each cycle, the suspension was stirred and the temperature was checked (not exceeding 38 °C). Upon completion of all cycles, the press cake was centrifuged (4000 rpm, 8 min) to a residual moisture content of 68–72% and immediately transferred to drying.

The selected protocol (5 min pulsed mode + pause) provided sufficient disruption of cell walls (porosity increase of 25–40% according to scanning electron microscopy data) while causing minimal losses of carotenoids and betalains (<3–5%).

Parameters were optimized to achieve maximum cell wall disruption without significant loss of bioactive compounds.

### 4.3. Drying Methods

Experiments included four drying schemes for each pomace type (carrot, beet, pumpkin). Convective drying (CD): performed in a Memmert UF110 drying oven (Memmer, Schwabach, Germany) at 55 ± 2 °C, air velocity 1.5–2.0 m/s, until final moisture content < 8%; US + CD: ultrasound-pretreated pomace was dried using the same CD scheme; and vacuum-microwave drying (VMD): carried out using a laboratory MagWave-3000 unit (ABB MagWave, Shanghai, China) with microwave power 300–600 W, pressure 5–15 kPa (vacuum 0.05–0.1 MPa), product temperature 30–60 °C. Drying was conducted in pulsed mode (on/off 30/10 s) until final moisture content was 5–6%; US + VMD: combination of ultrasound pretreatment and VMD using the specified parameters.

Optimization of the vacuum-microwave drying parameters (VMD and US + VMD) was performed using a full factorial experimental design (microwave power 200–600 W, pressure 5–15 kPa, pulsed mode duration). Optimization criteria (multi-criteria Harrington desirability function): maximum retention of key bioactive compounds (β-carotene in carrots ≥ 92%, betalains in beets ≥ 90%, total carotenoids in pumpkin ≥ 91%); minimum time to reach moisture content < 6%; minimum ΔE value (color change ≤ 10–12 units); low water activity (a_w_ ≤ 0.32); good Carr index (≤28–30%).

Optimal parameters for each raw material type: Carrots. Microwave power 400 W, pressure 8–10 kPa, pulsed mode 30 s on/10 s off, total time 90–140 min (US + VMD) → β-carotene retention 94–95%, ΔE = 9–11; Beets. Microwave power 300–350 W, pressure 5–8 kPa (deeper vacuum to minimize betalain oxidation), pulse 25 s on/15 s off, time 80–130 min → betalain retention 93–94%, ΔE = 8–10; Pumpkin. Microwave power 250–300 W (reduced due to high fiber content and risk of local overheating), pressure 10–12 kPa, pulse 35 s on/10 s off, time 110–170 min → carotenoid retention 92–93%, ΔE = 10–12.

### 4.4. Determination of Drying Kinetics

Drying kinetics were monitored by weighing samples every 5–10 min (for VMD every 2–5 min) on analytical balances Radwag AS 220.R2 (RADWAG, Radom, Poland) (accuracy 0.001 g). Moisture content was calculated using the formula:

W(t) = [(m_0_ − m(t))/(m_0_ − m_dry_)] × 100%,

where m_0_ is the initial mass, m(t) is the mass at time t, and m_dry_ is the dry mass (after oven drying at 105 °C to constant weight).

The effective moisture diffusivity coefficient (D_eff_) was determined using Fick’s second-order model. Model constants (Weibull, Midilli–Kucuk) were fitted by the least squares method in Statistica 13 software.

### 4.5. Physicochemical and Functional Properties of Powders

After drying, pomace was ground in a ball mill to particle size < 500 μm. The following properties were determined: particle size distribution was determined by laser diffraction using a Mastersizer 3000 analyzer (Malvern Panalytical, Malvern, UK) with dry dispersion; moisture content—AOAC 934.01; water activity (a_w_)—using an Aqualab 4TE hygrometer (Decagon Devices, Pullman, WA, USA); flowability—Carr’s index and Hausner ratio [46]; dispersibility and solubility—according to Cano-Chauca et al. (2005) [47]; color—using a Konica Minolta CR-400 colorimeter (Konica Minolta, Inc., Tokyo, Japan) (L, a, b, ΔE according to CIE Lab); thermal stability—heating of powders in a muffle furnace at 180–200 °C for 30 min, followed by determination of residual bioactive activity.

### 4.6. Determination of Bioactive Compounds

Total carotenoid and β-carotene content—spectrophotometrically (AOAC, Rockville, MD, USA, 2005) (UV-Vis spectrophotometer, Shimadzu UV-1800, Shimadzu Corporation, Kyoto, Japan) and by HPLC (Agilent 1260 Infinity, Agilent Technologies, Waldbronn, Germany, C18 column, detection at 450 nm). Betalains—spectrophotometrically [48]. Total antioxidant capacity—DPPH and FRAP methods.

### 4.7. Statistical Analysis

All experiments were performed in three replicates (n = 3). Data were processed using Statistica 13 and OriginPro 2021 software.

To assess the influence of two factors—raw material type (3 levels: carrot, beet, pumpkin) and drying method (4 levels: CD, US + CD, VMD, US + VMD)—on the studied parameters, a two-way analysis of variance (two-way ANOVA) was applied under the fixed effects model:

Y_ijk_ = μ + α_i_ + β_j_ + (αβ)_ij_ + ε_ijk_,

where α_i_ is the effect of raw material type (i = 1, …, 3), β_j_ is the effect of drying method (j = 1, …, 4), (αβ)_ij_ is the interaction effect, and ε_ijk_ is the random error.

Prior to post-hoc comparisons, the significance of the main effects and interaction was checked using the F-criterion (Fisher’s test). In all cases, differences were considered significant at *p* < 0.05.

When a significant interaction between raw material type and drying method was observed (*p* < 0.05 for the (αβ)_ij_ term), post-hoc comparisons of means were performed using Duncan’s multiple range test. These were conducted separately for each level of one factor while fixing the level of the other (simple main effects), or across the combinations of “raw material × drying method” (12 groups).

## 5. Conclusions

The studies have established that the combined method of ultrasound pretreatment with vacuum-microwave drying (US + VMD) demonstrates the best combination of kinetic, quality, and functional characteristics among all the approaches investigated. However, the effect of the method is not universal and varies significantly depending on the type of vegetable pomace and its rheological properties.

- For red beet pomace, the maximum relative drying acceleration is observed (up to 7–11× compared to convective drying), which is attributed to the high content of soluble sugars and nitrates. These components reduce the viscosity of the system and promote intense evaporation of free moisture under vacuum conditions.
- Pumpkin pomace exhibits the lowest acceleration (5.5–8×), primarily due to the high proportion of insoluble dietary fibers and pectic substances, which create additional diffusion barriers and slow down the removal of bound moisture even under combined intensive methods.
- For carrot pomace, the effect is intermediate (6–9×), with carotenoids exerting a noticeable influence on local changes in the dielectric properties of the material during vacuum-microwave heating.
- The differences in the degree of drying acceleration among the types of press cake highlight the need to account for the compositional features of the raw material (content of soluble substances, dietary fibers, pigments) when optimizing parameters, which is consistent with hypotheses regarding the influence of these components on mass and heat transfer.
- The superior retention of β-carotene (85–95 mg/100 g dw) and higher FRAP/DPPH values in US + VMD samples align with findings on reduced thermal degradation under vacuum-microwave conditions and cavitation-enhanced mass transfer from ultrasound pretreatment. These results are consistent with previous reports on improved bioactive preservation in microwave-vacuum dried vegetable pomace.

Thus, for the industrial implementation of technologies for deep processing of vegetable waste into functional powders, it is essential to take into account the type of raw material and its rheological characteristics (suspension viscosity, content of soluble/insoluble components, porosity after pretreatment, etc.). This will enable targeted optimization of drying parameters, minimization of energy consumption, and maximum retention of bioactive compounds and functional properties of the powders.

Such an approach will allow adaptation of the technology to specific types of vegetable waste, increase its economic efficiency, and contribute to sustainable valorization of waste in Kazakhstan.

## Figures and Tables

**Table 1:** Time to reach final moisture content <6% and relative drying acceleration for carrot, beet, and pumpkin pomace using different methods.

Pomace Type	Drying Method	Drying Time to <6% Moisture	Acceleration Relative to CD	Note/D_eff_ (Relative to CD)
Carrot	CD	600–840 min (10–14 h)	1× (baseline)	—
	US + CD	420–600 min (7–10 h)	1.4–1.5×	Acceleration due to microchannels
	VMD	120–180 min (2–3 h)	5–7×	Volumetric heating + vacuum
	US + VMD	90–140 min (1.5–2.3 h)	6–9×	Maximum acceleration (D_eff_ 3–5×)
Beet	CD	720–960 min (12–16 h)	1× (baseline)	—
	US + CD	480–720 min (8–12 h)	1.3–1.5×	Acceleration due to microchannels
	VMD	100–160 min (1.7–2.7 h)	6–9×	High sugar content accelerates
	US + VMD	80–130 min (1.3–2.2 h)	7–11×	Maximum acceleration (D_eff_ 3–5×)
Pumpkin	CD	660–900 min (11–15 h)	1× (baseline)	—
	US + CD	480–660 min (8–11 h)	1.3–1.4×	Acceleration due to microchannels
	VMD	140–200 min (2.3–3.3 h)	4.5–6.5×	High fiber content slows down
	US + VMD	110–170 min (1.8–2.8 h)	5.5–8×	Maximum acceleration (D_eff_ 3–5×)

**Table 2:** Physicochemical properties of powders.

Parameter	Carrot VMD	Carrot US + VMD	Beet VMD	Beet US + VMD	Pumpkin VMD	Pumpkin US +VMD	CD (Average Across Types)	US + CD (Average)
Moisture, %	5.6 ± 0.3	5.4 ± 0.2	5.2 ± 0.2	5.1 ± 0.1	6.1 ± 0.4	5.9 ± 0.3	7.8 ± 0.5	7.4 ± 0.4
a_w_	0.30 ± 0.02	0.29 ± 0.01	0.28 ± 0.01	0.27 ± 0.01	0.33 ± 0.02	0.31 ± 0.02	0.43 ± 0.04	0.39 ± 0.03
Carr’s index, %	30.4 ± 1.2	26.8 ± 1.0	23.7 ± 0.9	22.5 ± 0.8	29.3 ± 1.1	25.9 ± 0.9	35.2 ± 1.8	32.1 ± 1.5
Dispersibility (30 s), %	78 ± 3	85 ± 2	82 ± 3	88 ± 2	72 ± 4	80 ± 3	62 ± 5	68 ± 4

**Table 3:** Content of β-carotene, total phenolics (TPC), and antioxidant activity (DPPH and FRAP) in dried carrot, beetroot, and pumpkin pomace using different methods (mean ± SD, n = 3).

Pomace Type	Drying Method	β-Carotene (mg/100 g dw)	TPC (mg GAE/g dw)	DPPH (% Inhibition at 1 mg/mL)	FRAP (µmol TE/g dw)
Carrot	CD	45–55	8.5–10.5	65–75	180–220
	US + CD	65–75	11.0–13.5	78–85	240–280
	VMD	80–90	13.0–15.5	85–92	300–350
	US + VMD	85–95 (retention ~ 90%)	14.5–17.0	88–95	320–380
Beetroot	CD	— (focus on betalains)	12.0–15.0	70–80	220–260
	US + CD	—	15.0–18.0	80–88	280–320
	VMD	—	17.0–20.0	85–93	340–390
	US + VMD	—	18.5–22.0	88–96	360–410
Pumpkin	CD	25–35	6.0–8.0	55–65	140–180
	US + CD	35–45	8.0–10.0	68–78	190–230
	VMD	45–55	9.5–12.0	75–85	240–280
	US + VMD	50–60 (retention ~ 90%)	10.5–13.5	80–90	260–300

## Data Availability

The data presented in this study are available on request from the corresponding author.
